# Acquired Class D β-Lactamases

**DOI:** 10.3390/antibiotics3030398

**Published:** 2014-08-21

**Authors:** Nuno T. Antunes, Jed F. Fisher

**Affiliations:** Department of Chemistry and Biochemistry, University of Notre Dame, Notre Dame, IN 46556, USA; E-Mail: Jed.F.Fisher.57@nd.edu

**Keywords:** OXA, β-lactamase, plasmid, class D, carbapenemase, kinetics, crystal structure

## Abstract

The Class D β-lactamases have emerged as a prominent resistance mechanism against β-lactam antibiotics that previously had efficacy against infections caused by pathogenic bacteria, especially by *Acinetobacter baumannii* and the *Enterobacteriaceae*. The phenotypic and structural characteristics of these enzymes correlate to activities that are classified either as a narrow spectrum, an extended spectrum, or a carbapenemase spectrum. We focus on Class D β-lactamases that are carried on plasmids and, thus, present particular clinical concern. Following a historical perspective, the susceptibility and kinetics patterns of the important plasmid-encoded Class D β-lactamases and the mechanisms for mobilization of the chromosomal Class D β-lactamases are discussed.

## 1. Introduction

The β-lactams are one of the most efficacious antimicrobials used in human and animal clinical practice [1]. They kill bacteria by inactivating the “penicillin binding protein”, enzymes with essential roles in the synthesis of the bacterial cell wall, ultimately leading to cellular death [2,3]. The most important mechanism of resistance against the β-lactams in Gram-negative bacteria is their enzymatic inactivation by β-lactamases, enzymes that irreversibly open the β-lactam ring of antibiotics [4]. Based on amino acid sequence the β-lactamases are classified into four groups. The Class A, C, and D β-lactamases use an active site serine for opening of the β-lactam ring, while the Class B enzymes are metallo-β-lactamases [5]. Currently, more than one thousand β-lactamase variants are described and their number is increasing steadily [6].

Class D β-lactamases are also known as oxacillinase, or OXA, enzymes due to the fact that the first OXA enzymes described had a higher hydrolysis rate for the penicillin oxacillin than for benzylpenicillin, although this generalization is no longer valid [5,7]. Based on the percentage of increase in new enzymes and new enzyme variants, the OXA β-lactamase class has seen the largest recent growth [8]. Although this class contains a heterogeneous group of enzymes, they nonetheless may be grouped by amino acid identity. Their genes are found on both the chromosomes as well as the plasmids of diverse bacterial species such as *Acinetobacter*, *Shewanella*, *Pseudomonas*, and *Burkholderia* [9,10]. Many chromosomal Class D β-lactamases have now been transferred to plasmids, and pose a greater clinical threat [6,11]. Some OXA enzymes are limited in their substrate profile and only accept a narrow spectrum of substrates such as penicillins and first generation cephalosporins (narrow spectrum); others have expanded their spectrum of activity to include later generation cephalosporins (extended spectrum) and even carbapenem antibiotics (carbapenemases). In many groups of enzymes single amino acid substitutions are responsible for the expansion of this spectrum of activity, as seen in with OXA-2 and OXA-10. As the substrate spectrum of these enzymes did not include the carbapenems, their activity was not regarded as clinically significant until the discovery of the first carbapenem-hydrolyzing Class D β-lactamase (CHDL), OXA-23 (also known as ARI-1) [12]. Subsequent events demonstrate an inexorable increase in the number of CHDLs and their dissemination among some of the most difficult Gram-negative pathogens, including multidrug-resistant *Acinetobacter*, *Pseudomonas* and *Enterobacteriaceae* [7,10]. These opportunistic pathogens are an important contemporary source of nosocomial infections with high mortality [13]. As these enzymes are commonly associated with integrons, insertion sequences and transposons, they can be transferred between species [10,14]. Other β-lactamases now coexist with OXA enzymes on the same plasmid [10,15], resulting in a phenotypic spectrum of resistance that exceeds that of the individual enzymes. Moreover, their association with other resistance determinants such as to fluoroquinolones [16] and to aminoglycosides [17] has resulted in the co-selection of important resistance determinants. This review focuses on the distribution, substrate profile, known mutants, genetic environment, and structural studies of the OXA enzymes acquired by human pathogens.

## 2. General Properties

The Class D β-lactamase family is very diverse, with over 400 variants currently recognized [18]. This diversity is a source of confusion within the literature for the description of class D β-lactamases, especially at the level of amino acid identity. In order to provide a clearer organization to this family, we use a value of ≥80% amino acid identity to separate the Class D β-lactamase family into groups. Within each group we identify subgroups comprised of those enzymes having at least 95% amino acid identity. Although the selection of these cut-off values may be debated, we believe that these values suffice to more clearly establish the relationships among the diverse enzymes that comprise this family of enzymes. This manuscript describes the groups and sub-groups determined using this classification. Where different subgroups are present, we discuss separately the known clinical variants of each. 

Table 1 compares the amino acid identity of the fourteen OXA groups described in this review. Although the amino acid identity between them ranges between 17% to 75%, many have similar substrate and kinetic profiles. This similarity suggests the convergent evolution of resistance determinants from different origins.

**Table 1:** Amino acid identities between the different groups of Class D β-lactamases.

Group														
**Group**	**LCR-1**	**OXA-1**	**OXA-2**	**OXA-9**	**OXA-10**	**OXA-18**	**OXA-20**	**OXA-23**	**OXA-24/40**	**OXA-45**	**OXA-48**	**OXA-51**	**OXA-58**	**OXA-134**
LCR-1	-													
OXA-1	24	-												
OXA-2	35	24	-											
OXA-9	19	27	20	-										
OXA-10	30	22	34	20	-									
OXA-18	20	27	21	46	19	-								
OXA-20	37	22	75	19	33	20	-							
OXA-23	28	19	26	16	32	18	26	-						
OXA-24/40	29	21	25	16	35	18	26	59	-					
OXA-45	18	26	20	42	22	66	18	18	17	-				
OXA-48	34	24	39	17	46	23	40	34	31	21	-			
OXA-51	27	22	26	19	32	19	28	57	62	18	33	-		
OXA-58	31	22	28	18	33	19	28	47	48	18	31	50	-	
OXA-134	31	19	26	17	31	17	27	58	56	17	30	57	51	-
OXA-198	54	22	33	18	33	18	35	29	29	17	35	30	31	28

**Figure 1:**
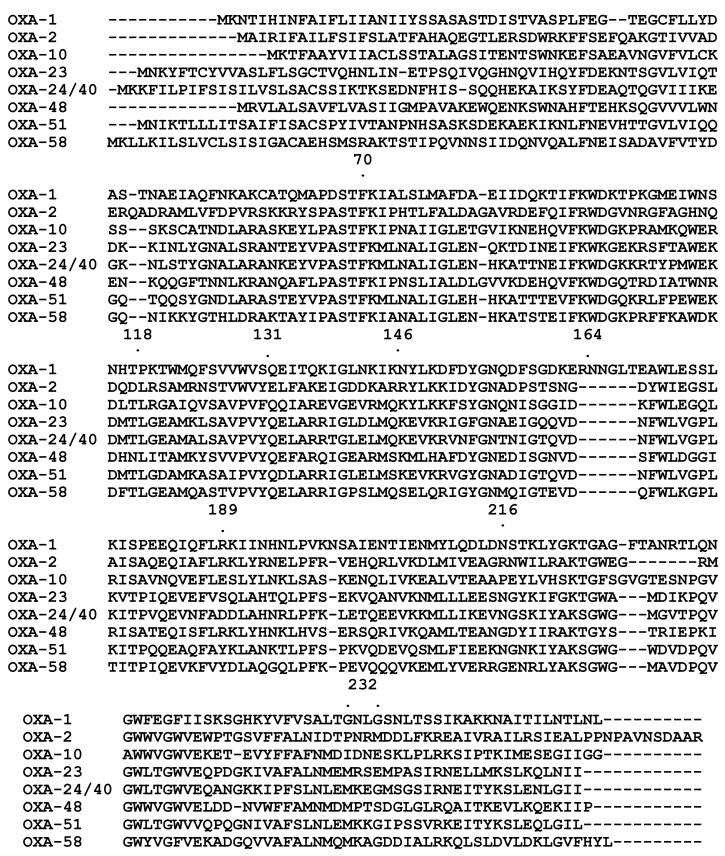
Amino acid alignment of seven Class D β-lactamases. Shaded in gray are motifs that are well conserved among this class of enzymes.

Despite this variability, all Class D β-lactamases display several conserved amino acid residues and motifs (Figure 1). The motif Ser-Thr-Phe-Lys that is present in most Class D β-lactamases is located at positions 70–73 (DBL numbering system), and is equivalent to a nearly identical motif that is found in Class A β-lactamases. However, exceptions occur among the chromosome-encoded OXA-62 and OXA-85 enzymes, which have a Ser-Thr-Tyr-Lys and Ser-Ser-Phe-Lys motif respectively [19,20]. A second conserved motif is Tyr-Gly-Asn (Phe-Gly-Asn in OXA-23 and OXA-24, and Phe-Gly-Glu in OXA-198) found in positions 144–146. The Lys-Thr-Gly (or Lys-Ser-Gly) motif in positions 216–218 is also highly conserved. Other conserved residues include Ser118, Gly131, Trp164, Leu189, Trp234 and Gly237.

## 3. Catalytic Mechanism

Notwithstanding the amino acid sequence diversity within the OXA Class D β-lactamases, and the yet further diversity in sequence seen upon comparison with the Class A and Class C β-lactamases, these three classes are mechanistically related to each other as serine-dependent β-lactamases [21]. Moreover, each of the three serine β-lactamase classes share topological and mechanistic similarities. The eponymous serine (Ser-67 in customary OXA numbering) of the OXA β-lactamases is the active site nucleophile used during enzymatic catalysis. This serine adds to the carbonyl of the β-lactam antibiotic to initiate turnover. The addition intermediate collapses subsequently, with the concurrent departure of the nitrogen of the β-lactam, to form an acyl-enzyme intermediate [22]. Turnover is completed by a second reaction wherein a water molecule is itself activated as a nucleophile to add to the carbonyl carbon of the acyl-enzyme intermediate, and subsequent collapse of this intermediate to restore the free serine and the hydrolyzed (inactivated) β-lactam antibiotic. For most substrates of the serine β-lactamases, the initial acylation of the serine is the slow step in catalysis. For many inhibitors of the serine β-lactamases—notably clavulanate, tazobactam, avibactam, and the bicyclic β-lactam inhibitors—the ability of these inhibitors to form an acyl-enzyme species, but then to have this acyl-enzyme resistant to catalytic hydrolysis—is foundational to their ability as β-lactamase inhibitors.

The distinguishing feature of the three serine β-lactamase classes is the mechanistic basis by which their active site serine is activated as a nucleophile for acylation, and water is activated as a nucleophile for deacylation. Both events require a catalytic residue to facilitate proton movement during catalysis. Each of the three β-lactamase classes uses different catalytic residue(s) to this purpose. The Class A β-lactamases use a glutamate-lysine pair as the key catalytic residues activating the serine during acylation, and water during deacylation. The catalytic residues for the Class C β-lactamases are a lysine-tyrosine pair [23]. For the Class D β-lactamases, the key catalytic residue is a carbamate anion formed by a spontaneous post-translational modification wherein carbon dioxide reacts with the β-amine of an active site lysine [24,25,26,27]. The carbamate anion formed in this reaction acts as the so-called general base that accepts a proton from serine, activating the serine for acyl-enzyme formation to initiate turnover; and accepts a proton from water, activating the water as a nucleophile for acyl-enzyme hydrolysis to complete turnover. In the absence of carbamate formation at the ɛ-amine of the active site lysine, the OXA Class D β-lactamase is catalytically inactive. This simple correlation—catalytic activity following reaction of the active site lysine with carbon dioxide, loss of catalytic activity in the absence of this reaction—has confounded *in vitro* kinetic study of these enzymes in the past and even to today. Simple experimental circumstances (notably acidic pH, or the spontaneous decarboxylation that can occur during enzyme purification, handling, and storage) result in loss of the requisite carbamate anion required for catalysis. Reliable and reproducible *in vitro* OXA kinetics are obtained *only* when the enzyme is provided a carbon dioxide source (customarily, as bicarbonate anion) both at the start and during the course of the kinetic study. Reliable experimental procedures that address this requirement are implemented trivially (for example, see reference [28]) but are not always used. In the data compilations presented in the following sections attention is called to those studies where a critical comparison of the kinetic data is not possible for this reason.

## 4. MIC and Kinetic Characteristics

Table 2 gives the MIC values for representative β-lactams for representative members of eleven OXA groups, each expressed in the identical *E. coli* vector. In general, all OXA enzymes except OXA-69 (an OXA-51 derivative) confer resistance to penicillins, with the exception of the ureidopenicillin piperacillin for which MIC values can be of susceptibility or resistance. OXA-69 does not raise significantly the MICs for any of the tested β-lactams [29]. OXA enzymes, with the exception of OXA-10 and OXA-18, have a limited ability to decrease the susceptibility of extended-spectrum cephalosporins and aztreonam, a monobactam. An important caveat is that these MIC values are obtained using the first enzyme of each group to be described. Several point mutation-variants of each enzyme showing an expanded spectrum of activity (and hence clinically significant resistance, as discussed later) are found in clinical isolates. Only a few of the known Class D β-lactamases increase the MICs for carbapenems.

## 5. Kinetics

OXA enzymes were originally called oxacillinases because they hydrolyze oxacillin at a rate at least 50% higher than the rate for benzylpenicillin [30]. An updated classification has been suggested for the OXA enzymes which uses the substrate and inhibitor profile of each [31]. Although literature references using this new classification are common, we do not use it here due to the fact that much of the kinetic data used for the classification are suspect, due to the experimental failure to provide a source of carbon dioxide during the kinetic determinations. The absence of a CO_2_ source significantly alters the kinetic data [24,32]. Examples of the different kinetic data, in the absence (plain font) and presence of CO_2_ (bold font), are given in Table 3. Kinetic data obtained with a CO_2_ source cannot be meaningfully compared to kinetic data obtained without a CO_2_ source. The latter type of kinetic data is found frequently in the literature.

Class D β-lactamases effectively hydrolyze penicillins and early generation cephalosporins such as cephalothin, while poorly hydrolyzing extended spectrum cephalosporins and aztreonam. The hydrolysis of carbapenems is also slow, with imipenem usually hydrolyzed faster than either meropenem or doripenem (with the exception of OXA-2 and -10). However, many of the OXA enzymes demonstrate very high affinity—often in the nanomolar range—for carbapenems [28]. The remarkable effect of this affinity is seen for the MICs obtained with an *Acinetobacter* or *Pseudomonas* background [28,29,30,33]. These organisms accumulate a remarkably low amount of foreign substrates in their cytoplasm as a result of the combination of decreased permeability and the presence of multiple efflux systems. Indeed, the MICs determined in a background strain with over-expressed efflux pumps or with down-regulated porins are even higher (Table 4) [29,34,35]. The acquisition of OXA β-lactamases with exceptionally high affinity by bacteria with low permeability may be the reason for the clinical challenge presented by these bacteria. While the kinetic data alone would seem inadequate to support carbapenem resistance, the observed susceptibility patterns represent a successful balance among permeability, enzyme catalytic properties, and the amount of enzyme in the periplasm [28].

**Table 2:** Minimal inhibitory concentrations for selected Class D β-lactamases in *E. coli* background.

MIC (µg/mL)															
Antimicrobial	*E. Coli* JM83	OXA-2	OXA-10	OXA-23	OXA-24/40	OXA-48	OXA-58	*E. Coli* DH10B ^a^	OXA-1	OXA-69	*E. Coli* TOP10	OXA-134	*E. Coli* JM109	OXA-18	OXA-20
Benzylpenicillin	16	1024	4096	4096	2048	512	2048	-	-	-	-	-	-	-	-
Ampicillin	2	1024	16384	4096	2048	256	2048	4	-	16	-	-	-	-	-
Amoxicillin	4	1024	>2048	>2048	2048	512	2048	4	>512	-	4	>512	2	32	512
Oxacillin	256	2048	8192	8192	1024	1024	4096	-	-	-	-	-		-	-
Ticarcillin	4	4096	32,768	16,384	16,384	8192	16384	4	>512	8	4	>512	2	128	512
Piperacillin	1	128	512	256	128	16	512	4	128	4	1	8	1	16	4
Cephalothin	4	64	64	32	8	8	32	2/4	8	4	2	8	4	8	32
Cefuroxime	4	8	128	8	4	4	4	4	8	4	2	4	-	-	-
Cefoxitin	2	4	8	2	2	2	2	-	-	-	-	-	8	8	16
Cefmetazole	1	2	8	1	1	1	1	-	-	-	-	-	-	-	-
Moxalactam	0.5	1	16	4	2	2	16	0.12	0.12	-	0.06	0.12	0.25	1	0.25
Ceftazidime	0.12	8	4	0.12	0.12	0.12	0.12	0.25/0.06	0.25	0.06	0.06	0.12	0.25	64	0.5
Cefotaxime	0.03	0.12	2	0.25	0.03	0.12	0.06	<0.06	0.12	-	0.12	0.12	0.06	2	0.06
Ceftriaxone	0.03	0.06	4	0.12	0.03	0.06	0.06	-	-	-	-	-	-	-	-
Cefepime	0.016	0.06	2	0.5	0.06	0.03	0.03	<0.06	0.5	0.06	0.06	0.12	0.06	1	0.12
Aztreonam	0.06	0.12	16	0.12	0.12	0.06	0.06	0.12	0.12	-	0.12	0.12	0.125	64	0.25
Imipenem	0.12	0.25	0.25	0.5	1	0.5	0.5	0.5	0.5	-	0.06	0.5	0.06	0.06	0.25
Meropenem	0.016	0.06	0.12	0.25	0.5	0.06	0.06	-	-	-	0.06	0.5	-	-	-
Ertapenem	0.004	0.12	0.5	0.5	0.5	0.12	0.06	-	-	-	-	-	-	-	-
Doripenem	0.03	0.12	0.12	0.25	0.5	0.12	0.06	-	-	-	-	-	-	-	-
Reference	[28]	[28]	[28]	[28]	[28]	[28]	[28]	[29,36]	[36]	[29]	[37]	[37]	[38]	[38]	[39]

^a^: when the MIC values for a certain antimicrobial against the control strain were different between the different references, we have included the two values so that comparisons could be made. The first value would be the MIC obtained when testing OXA-1 and the second when testing OXA-69.

**Table 3:** Kinetic values of several Class D β-lactamases against representative β-lactam antibiotics.

Enzyme	Benzylpenicillin	Ampicillin	Oxacillin	Cephalothin	Cefepime	Ceftazidime	Aztreonam	Imipenem	Meropenem	Doripenem	Ref.
OXA-1	ND	**520 ± 6**	NDND	**2.7 ± 0.1**	**34 ± 1**	ND	ND	ND	ND	ND	[40]
*k*_cat_ (s^−1^)*K*_m_ (µM)	ND	**21 ± 1**	ND	**9 ± 0.7**	**170 ± 14**	ND	ND	ND	ND	ND	
OXA-2	ND	ND	**1100 ± 30**	ND	ND	ND	ND	**0.18 ± 0.01**	**0.11 ± 0.01**	**0.20 ± 0.01**	[28]
*k*_cat_ (s^−1^)*K*_m_ (µM)	ND	ND	**210 ± 20**	ND	ND	ND	ND	**≤2.0**	**≤2.0**	**≤2.0**	
OXA-10	**109 ± 3**	**143 ± 7**	**530 ± 10**	**8.3 ± 0.1**	ND	ND	ND	**0.041 ± 0.001**	**0.039 ± 0.001**	**0.037 ± 0.001**	[24,28]
*k*_cat_ (s^−1^)*K*_m_ (µM)	**23 ± 0.4**	**34 ± 4**	**87 ± 5**	**32 ± 2**	ND	ND	ND	**≤2.0**	**5.6 ± 0.8**	**4.8 ± 0.8**	
OXA-20	26 ± 0.9	80 ± 1.4	116 ± 5	13 ± 0.05	ND	ND	6.0 ± 0.5	ND	ND	ND	[39]
*k*_cat_ (s^−1^)*K*_m_ (µM)	4.4 ± 1.3	33 ± 2.2	329 ± 34	5 ± 0.2	ND	ND	69 ± 12	ND	ND	ND	
OXA-23	**460 ± 10**	ND	**320 ±20**	ND	ND	**<0.01**	**0.24 ± 0.01**	**0.35 ± 0.01**	**0.068 ± 0.001**	**0.036 ± 0.001**	[28,41]
*k*_cat_ (s^−1^)*K*_m_ (µM)	**82 ± 9**	ND	**110 ± 10**	ND	ND	**ND**	**2400 ± 140**	**≤2.0**	**≤2.0**	**≤2.0**	
OXA-24/40	**480 ± 20**	5	**170 ± 10**	3	<0.001	**<0.01**	**ND**	**1.7 ± 0.1**	**0.11 ± 0.01**	**0.084 ± 0.001**	[28,41,42]
*k*_cat_ (s^−1^)*K*_m_ (µM)	**180 ± 20**	220	**ND**	72	ND	**ND**	**>3000**	**≤2.0**	**≤2.0**	**≤2.0**	
OXA-48	245	340	**160 ± 10**	3	1	4	ND	**6.7 ±0.2**	**0.16 ± 0.01**	**0.14 ± 0.01**	[28,43]
*k*_cat_ (s^−1^)*K*_m_ (µM)	40	5,200	**≤30**	20	160	5,100	ND	**≤2.0**	**≤2.0**	**≤2.0**	
OXA-58	**59 ± 8**	**88 ± 14**	**160 ±10**	**19 ± 3**	<0.01	≤2.0	<0.01	**1.8 ±0.1**	**0.019 ± 0.001**	**0.014 ± 0.001**	[28,32,44]
*k*_cat_ (s^−1^)*K*_m_ (µM)	**11 ± 2**	**77 ± 8**	**53 ± 6**	**190 ±30**	ND	ND	ND	**6.0 ±0 0.5**	**≤2.0**	**≤2.0**	
OXA-69	**0.2**	**0.06**	**0.2**	**0.004**	ND	ND	<0.01	**0.1**	**0.06**	**MD**	[29]
*k*_cat_ (s^−1^)*K*_m_ (µM)	**710**	**240**	**3700**	**190**				**3600**	**4500**		
OXA-134	70	150	ND	ND	<0.01	<0.01	<0.01	0.1	0.05	ND	[37]
*k*_cat_ (s^−1^)*K*_m_ (µM)	50	250	ND	ND	<0.01	<0.01	<0.01	10	250	ND	

The data in bold represent values obtained in the presence of saturating amounts of carbon dioxide; ND: not determined.

**Table 4:** MICs for OXA-58 in different background strains (adapted from [37]).

Antimicrobial	MIC (µg/mL)					
	*E. coli* DH10B	OXA-58	*A. baumannii* CIP70.10	OXA-58	*A. baumannii* BM4547 ^a^	OXA-58
Ticarcillin	4	>256	4	>256	8	>256
Ceftazidime	0.06	0.06	2	2	4	4
Imipenem	0.06	0.25	0.25	0.5	0.5	1

^a^: This strain is a point mutant of *A. baumannii* CIP70.10 over-expressing the AdeABC efflux pump. Note that the MIC values are lower than expected in the *A. baumannii* transformants. The explanation for this values may be the use of laboratorial strains and weaker promoters than the ones found in nature.

## 6. Inhibitors

Class D β-lactamases are classically described as poorly inhibited by the β-lactamase inactivators clavulanic acid, tazobactam and sulbactam. This description may be a misconception. Table 5 shows the variations in the MICs of several penicillins in the presence of these inhibitors when the OXA enzymes are expressed in *E. coli*. Tazobactam and clavulanic acid both show inhibitory activity for some, but not all, OXA enzymes. Some of the MIC values would classify the strains harboring these enzymes as susceptible to tazobactam. Recent studies demonstrate that OXA-10 is poorly inhibited by the new inhibitor avibactam, while OXA-48 is inhibited [45,46].

IC_50_ values determined for inhibitors (including sodium chloride) are shown in Table 6 (note, however, that these kinetic data were obtained without CO_2_ supplementation). Although firm conclusions cannot be drawn from the comparison of IC_50_ values, a distinct pattern emerges with respect to enzyme which correlates with the MIC data. Sodium chloride is a known inhibitor of Class D β-lactamases, although not all are susceptible (exemplified by OXA-24/40). NaCl inhibition has been associated with the presence of a tyrosine residue at position 144 (DBL numbering system), which is not present in both OXA-24/40 and -23. In fact, site directed mutagenesis replacement of the tyrosine by a phenylalanine restores that resistance [41].

## 7. Plasmid-Encoded Class D β-Lactamases

### 7.1. OXA-1

The OXA-1 enzyme is also the OXA-30 enzyme, as a result of error in the original OXA-1 sequence [47]. OXA-1 shares less than 30% of homology with other plasmid and chromosomal Class D β-lactamases.

*Distribution and hosts.* OXA-1-like enzymes are found in several bacterial species such as *P. aeruginosa*, *K. pneumoniae* and other *Enterobacteriaceae*, and in both human and animal isolates [11,30,43]. OXA-1-like enzymes have been described in Europe, Asia and Africa [30,43,48,49,50].

*Antibiotic susceptibility profile and substrate profile.* The wild-type OXA-1 enzyme is a narrow spectrum β-lactamase that confers resistance to penicillins and decreases the susceptibility to cephalothin, cefotaxime and cefepime. The MICs of carbapenem antibiotics and ceftazidime remain unchanged. It is not sensitive to tazobactam. Kinetics made with partially purified enzyme demonstrated that OXA-1 effectively hydrolyzes penicillins and cephalosporins [30].

**Table 5:** MICs for penicillins in the presence and absence of inhibitors against diverse enzymes.

Antimicrobial	OXA-1	OXA-2	OXA-10	OXA-18	OXA-20	OXA-23	OXA-24	OXA-48	OXA-58	OXA-69	OXA-134
Amoxicillin	>512	>512	-	32	512	>1024	>512	>512	>512	-	>512
Amoxicillin + clavulanic acid ^a^	128	64	-	4	32	>1024	>512	>512	128	-	128
Ticarcillin	>512	>512	>512	128	512	>1024	>512	>512	>512	8	>512
Ticarcillin + clavulanic acid ^a^	256	32	128	8	32	1024	>512	>512	256	8	256
Piperacillin	128	64	128	16	4	32	256	128	8	4	8
Piperacillin + tazobactam ^b^	128	2	32	1	1	4	128	128	8	4	8
Reference	[41]	[18]	[51]	[38]	[39]	[52]	[41]	[43]	[44]	[29]	[37]

^a^: Clavulanic acid held constant at 2 µg/mL; ^b^: Tazobactam held constant at 4 µg/mL. AMX: amoxicillin; CLA: clavulanic acid; TI: ticarcillin; PIP: piperacillin; TZB: tazobactam.

**Table 6:** IC_50_ values for selected β-lactamases.

Inhibitor	IC_50_							
	OXA-2	OXA-10	OXA-18	OXA-20	OXA-24/40	OXA-48	OXA-58	OXA-69
Clavulanic acid (µM)	3	<40	0.08	2.2	300	16	310	100
Tazobactam (µM)	0.05	-	0.123	-	180	1.7	60	40
Sulbactam (µM)	0.1	<40	0.56	-	190	50	2500	30
NaCl (mM)	-	19	-	-	3000	7	12	7.5
Reference	[18]	[53]	[38]	[39]	[41]	[43]	[44]	[29]

*Reported clinical mutants.* There are five sequences of OXA-1-like enzymes (Table 7) which share at least 95% amino acid identity. OXA-31 was identified in a multi-drug resistant French isolate of *P. aeruginosa* in 1999 in a non-transferrable plasmid. It differs from OXA-1 in three amino acids, but has identical susceptibility and kinetic profiles [30]. Another plasmid-born variant, OXA-47, was initially identified in Turkey in a *K. pneumoniae* isolate that also expressed OXA-48. The sequence of OXA-47 differs from OXA-1 in seven amino acids. OXA-47 confers resistance to amoxicillin and ticarcillin and decreased susceptibility to piperacillin, but does not change the susceptibility to carbapenems and cephalosporins. MICs obtained in the presence of inhibitors show an increased susceptibility to clavulanic acid but not to tazobactam [43]. 

**Table 7:** OXA-1 like enzymes.

β-Lactamase	Subgroup	Spectrum	First Reported Host	Chromosome/Plasmid	GenBank Accession Number	Ref.
OXA-1/30	OXA-1	Narrow	*E. coli*	Plasmid	AF255921	[50]
OXA-4	OXA-1	Narrow	*C. freundii*	Plasmid	AY162283	[54]
OXA-31	OXA-1	Expanded	*P. aeruginosa*	Plasmid	AF294653	[30]
OXA-47	OXA-1	Narrow	*K. pneumoniae*	Plasmid	AY237830	[43]
OXA-224	OXA-1	-	*P. aeruginosa*	Plasmid	JN596991	[55]

*Location and genetic environment.* Analysis of the genetic environment of several OXA-1-like enzymes shows the encoding gene to be associated with Class 1 integrons [11]. The OXA-1 gene was surrounded by an integrase gene and by an aminoglycoside acetyltransferase gene [56]. A very similar arrangement was observed for the gene encoding OXA-31 [30,57]. The genes for OXA-4 and -224 were also located associated with a Class 1 integron, surrounded by an aminoglycoside acetyltransferase and *qacEΔ1* [54,57]. A different gene organization is described for the gene encoding OXA-47 [43].

*Structure determination.* The crystal structure of the apoenzyme of OXA-1 was determined in 2003 [57], and later as the complex with doripenem [58]. A deacylation-deficient OXA-1 mutant structure is also described as apoenzyme and in complex with oxacillin [59,60].

### 7.2. OXA-2

The OXA-2 β-lactamase can be traced to the 1970s. The enzyme is characterized by a hydrolysis rate for oxacillin several times higher than for benzylpenicillin [61].

*Distribution and hosts.* OXA-2-like enzymes are described worldwide [62,63,64,65,66] and are predominantly, but not exclusively, found in *P. aeruginosa*. Other species in which OXA-2 like enzymes have been found are *Salmonella* [64], *K. pneumoniae* [67], *Acinetobacter* [68] and also the Gram-positive *Corynebacterium amycolatum* [10], amongst other species [10].

*Antibiotic susceptibility and substrate profiles.* Enzymes from this group were regarded as narrow-spectrum enzymes [10], but recent experiments demonstrate that this enzyme is in fact a carbapenemase [28]. When cloned into *E. coli* it confers resistance to penicillins and decreases the susceptibility to cephalothin, ceftazidime, and carbapenems. In *A. baumannii* background OXA-2 confers resistance to carbapenems with MIC values are very similar to the ones obtained for OXA-58 when tested under the same conditions [28]. Changes in the MICs determined in the presence of clavulanic acid and tazobactam show that this enzyme is susceptible to their inhibitory activity [18,63]. Kinetically, it behaves like a classical CHDL, with low turnover rates for carbapenems but with very high affinities. Its turnover rate for oxacillin is higher than the one obtained for other CHDLs [28]. The IC_50_ values for sulbactam, tazobactam and clavulanic acid are reported as 0.1, 0.05 and 3 μM, respectively [19]. 

*Reported clinical mutants.* Thirteen OXA-2-like enzymes are described, each sharing at least 80% amino acid identity (Table 8). A cutoff of 95% amino acid identity identifies four subgroups. Although many of the isolates are only clinical findings, some have been studied sufficiently to allow their classification as narrow or expanded-spectrum enzymes.

**Table 8:** OXA-2 like enzymes.

β-Lactamase	Subgroup	Spectrum ^a^	First Report Host	Chromosome/Plasmid	GenBank Accession Number	Ref.
OXA-2	OXA-2	Carbapenemase	*S. Typhimurium*	Plasmid	X07260	[68,69]
OXA-3	OXA-3	Narrow	*P. aeruginosa*	Plasmid	L07945	[9]
OXA-15	OXA-2	Expanded	*P. aeruginosa*	Plasmid	U63835	[63]
OXA-21	OXA-3	-	*A. baumannii*	Plasmid	Y10693	[70]
OXA-32	OXA-2	Expanded	*P. aeruginosa*	Plasmid	AF315351	[18]
OXA-34	OXA-2	-	*P. aeruginosa*	-	AF350424	-
OXA-36	OXA-2	-	*P. aeruginosa*	-	AF300985	-
OXA-46	OXA-46	Narrow	*P. aeruginosa*	Plasmid	AF317511	[71]
OXA-53	OXA-53	Expanded	*S. enterica*	Plasmid	AY289608	[72]
OXA-141	OXA-2	-	*P. aeruginosa*	-	EF552405	-
OXA-144	OXA-2	-	*A. baumannii*	-	FJ872530	-
OXA-161	OXA-2	Expanded	*P. aeruginosa*	Plasmid	GQ202693	[65]
OXA-210	OXA-2	-	*P. aeruginosa*	-	JF795487	-

^a^: OXA-2 is now known to be a carbapenemase [27]. The spectrum classification for the other enzymes of this group may need to be revised.

OXA-15 is an Asp150Gly mutant found on a transferrable plasmid in a *P. aeruginosa* strain isolated in Turkey in 1992 that had a considerable resistance to ceftazidime. When cloned into *E. coli* it decreased the susceptibility to ampicillin, ceftazidime, moxalactam and aztreonam, but did not change the MICs for carbenicillin and other cephalosporins. Kinetics performed with a semi-purified enzyme showed that it had an increased activity against ceftazidime and decreased activity against ampicillin when compared to OXA-2 [63]. OXA-32, a Leu164Ile mutant, was described in a ceftazidime-resistant *P. aeruginosa* isolate from the French West Indies. The gene was located on a transferrable plasmid. *E. coli* cells expressing the gene were less sensitive to ceftazidime, cefotaxime, cefepime, aztreonam, moxalactam, clavulanic acid and tazobactam, but more sensitive to amoxicillin, ticarcillin and cephalothin. It hydrolyzed several penicillins and cephalosporins, with the exception of cefotaxime [18]. OXA-161, an Asn148Asp single mutant of OXA-2, was described in a *P. aeruginosa* isolate from Spain showing an unusual resistance phenotype. When expressed in *P. aeruginosa* this mutant conferred less protection to carbenicillin than OXA-2, but the MICs to ceftazidime, aztreonam and cefepime were higher [65].

Although OXA-46 shares only 80.5% amino acid identity with OXA-2, we include it in the OXA-2 group. It was identified in a multi-drug-resistant *P. aeruginosa* isolate from Italy. When cloned in *E. coli*, it increased the MICs for penicillins and cephalothin, but not for carbapenems or extended-spectrum cephalosporins. No activity was seen against extended-spectrum cephalosporins or aztreonam [69].

*Location and genetic environment.* The genes encoding several OXA-2-like enzymes are associated with Class 1 integrons. The one for OXA-15 was located in a *sulI*-associated type transposon, between an aminoglycoside nucleotidyltransferase (*aadB*) and *qacEΔ1*, an ethidium bromide and quaternary ammonium resistance determinant [63]. OXA-32 was also located in a Class 1 integron, and was flanked by an integrase gene and an aminoglycoside acetyltransferase [18]. A very similar arrangement was observed for the genes of OXA-161 and OXA-53 [65,72]. The arrangement around the gene encoding OXA-46 was different; it was located in an integron containing two copies of *aacA4* and one of VIM-1 [69].

*Structure determination.* The crystal structure for the OXA-2 wild-type enzyme was determined (PDB access number 1K38) but has not yet been published. The structure of OXA-46 is described [73].

### 7.3. OXA-10

This enzyme was originally found in *Pseudomonas* and called PSE-2 [74].

*Distribution and hosts.* OXA-10-like enzymes have been identified in several human and animal isolates worldwide [46,66,75,76,77,78]. Although they are commonly associated with *P. aeruginosa*, they have also been reported in *A. baumannii* [79] and in the *Enterobacteriaceae* [75,80].

*Antibiotic susceptibility and substrate profiles.* When cloned into *E. coli*, OXA-10 conferred resistance to all tested penicillins and to cephalothin. It increased the MICs of expanded-spectrum cephalosporins, aztreonam and carbapenems, without however reaching clinically relevant levels. When cloned in *A. baumannii* it behaved as an expanded spectrum β-lactamase conferring resistance to carbapenems [28]. The enzyme efficiently hydrolyzes penicillins, cephalosporins and (at a low rate) carbapenems [24,28]. When compared with the other Class D carbapenemases, the carbapenem kinetics of OXA-10 do not correlate to a significant increase in the MIC values produced by the enzymes. This increase is attributed to greater amount of the OXA-10 enzyme in the periplasm [28].

*Reported clinical mutants.* Currently there are twenty-three clinical OXA-10-like enzymes with a widespread distribution in terms of host and geographic localization (Table 9). Although there are limited data for each, they are traditionally characterized as either narrow spectrum or expanded-spectrum [11]. OXA-11, a two amino acid mutant of OXA-10, was first described on a transferable plasmid in a Turkish isolate of *P. aeruginosa* isolated in 1991. The enzyme conferred higher MICs for cephalosporins, moxalactam and aztreonam than those conferred by OXA-10. Preliminary kinetics indicate that those increases may be predominantly the result of increased relative affinity for the antibiotic [81].

**Table 9:** OXA-10 like enzymes.

β-Lactamase	Subgroup	Spectrum	First Report Host	Chromosome/Plasmid	GenBank Accession Number	Ref.
OXA-10	OXA-10	Narrow ^a^	*P. aeruginosa*	Plasmid	U37105	[82]
OXA-5	OXA-5	Narrow	*P. aeruginosa*	Plasmid	X58272	[83]
OXA-7	OXA-10	Narrow	*E. coli*	Plasmid	X75562	[80]
OXA-11	OXA-10	Expanded	*P. aeruginosa*	Plasmid	Z22590	[81]
OXA-13	OXA-10	Expanded	*P. aeruginosa*	Chromosome	U59183	[77]
OXA-14	OXA-10	Expanded	*P. aeruginosa*	Plasmid	L38523	[76]
OXA-16	OXA-10	Expanded	*P. aeruginosa*	Plasmid	AF043100	[84]
OXA-17	OXA-10	Expanded	*P. aeruginosa*	Plasmid	AF060206	[85]
OXA-19	OXA-10	Expanded	*P. aeruginosa*	Plasmid	AF043381	[86]
OXA-28	OXA-10	Expanded	*P. aeruginosa*	Plasmid	AF231133	[87]
OXA-35	OXA-10	Expanded	*P. aeruginosa*	Chromosome	AF315786	[51]
OXA-56	OXA-10	Narrow	*P. aeruginosa*	-	AY445080	[88]
OXA-74	OXA-10	-	*P. aeruginosa*	Chromosome	AJ854182	[89]
OXA-101	OXA-10	-	*C. freundii*	Plasmid	AM412777	[75]
OXA-128	OXA-10	-	*A. baumannii*	-	EU375515	[79]
OXA-129	OXA-10	-	*S. enterica*	Plasmid	AM932669	
OXA-142	OXA-10	-	*P. aeruginosa*	-	EU358785	-
OXA-145	OXA-10	Expanded	*P. aeruginosa*	Chromosome	FJ790516	[90]
OXA-147	OXA-10	Expanded	*P. aeruginosa*	-	FJ848783	[91]
OXA-183	OXA-10	-	*P. aeruginosa*	-	HQ111474	-
OXA-240	OXA-10	-	*P. aeruginosa*	-	JX089628	-
OXA-251	OXA-10	-	*P. aeruginosa*	-	JN118546	-
OXA-256	OXA-10	-	*E. cloacae*	Plasmid	HE616889	[92]

^a^: OXA-10 gives MIC values similar to those obtained with other carbapenemases when expressed in *A. baumannii*.

OXA-14, a Gly157Asp mutant of OXA-10, was isolated in *P. aeruginosa* in Turkey in 1991. Its susceptibility profile to most tested β-lactams was similar to OXA-10, but OXA-14 was several-fold more resistant to cefepime and ceftazidime [76]. OXA-16 has two amino acid mutations compared to OXA-10. It was isolated in Turkey in 1993 in a clinical isolate of *P. aeruginosa*. OXA-16 increases the MICs of ceftazidime, cefotaxime, ceftriaxone, cefepime and moxalactam as compared to OXA-19 [84]. OXA-17 was identified in a *P. aeruginosa* isolate from Turkey and differs from OXA-10 by an Asn73Ser substitution. When cloned into *E. coli* its susceptibility pattern β-lactams was very similar to OXA-10: it increased the MICs for penicillins, carbenicillin, cefotaxime, ceftriaxone, cefepime, cefoperazone, moxalactam and aztreonam, but failed to increase MICs for imipenem and ceftazidime [85].

OXA-13 was isolated from a multi-drug resistant *P. aeruginosa* French isolate. It is considered an expanded-spectrum enzyme. It differs from OXA-10 by nine amino acids. OXA-13 increased the MICs of penicillins, cefoperazone, cefotaxime, ceftazidime and aztreonam, but did not change the MIC of imipenem [77]. OXA-19 differs from OXA-13 by two amino acids. It was isolated in France in 1991 from a *P. aeruginosa* strain that was highly resistant to ceftazidime. OXA-19 is less susceptible to penicillins than OXA-13, although the MICs of ceftazidime are nearly identical [86]. OXA-28 is a two amino acid mutant of OXA-13, identified in *P. aeruginosa*. It increased the MICs of penicillins, cefotaxime and aztreonam. Resistance to ceftazidime was observed [87]. OXA-35 was isolated in France in 1999 in *P. aeruginosa* and differs in eight amino acids from OXA-10, but only one from OXA-13. It has almost identical susceptibility and kinetic profiles to β-lactams as OXA-10, with the exception of a higher susceptibility to piperacillin [51,86]. OXA-145, an OXA-35 derivative, was isolated in the Reunion Islands in 2008. It differs from OXA-35 by a Leu155 deletion. It is more susceptible to penicillins than OXA-35, but is more resistant to extended spectrum cephalosporins and aztreonam [86]. A Trp154Leu mutation of OXA-35 gives OXA-147. This enzyme had an increased spectrum of activity against cephalosporins, while becoming more susceptible to penicillins [87]. Both enzymes were initially identified in *P. aeruginosa*.

*Location and genetic environment.* The genes encoding OXA-10 like enzymes are commonly found associated with Class 1 integrons and in some cases, the genes for OXA-35, OXA-145 and OXA-147 are downstream of an aminoglycoside acetyltransferase [51,86,87].

*Structure determination.* Multiple OXA-10 structures have been determined at different pH values, and show different levels of carboxylation of the active site lysine [24,26,93]. The structures of OXA-10 Tyr154 mutants have been determined as the apoenzyme and in complex with benzylpenicillin [94]. The structure of a clinical mutant, OXA-13, was determined as the apoenzyme and in complex with meropenem [95].

### 7.4. OXA-23

The first acquired CHDL was described in 1993 in a Scottish clinical *Acinetobacter baumannii* strain, first isolated in 1985. The CHDL OXA-23 (formerly known as ARI-1, for *Acinetobacter* Resistant to Imipenem) was located on a plasmid [13].

*Distribution and hosts.* OXA-23 like enzymes are found currently worldwide [11,16,96,97,98,99] in both human and animals isolates [100]. The origin of OXA-23 was traced to *Acinetobacter radioresistens*, a commensal skin bacterium, where its contribution to carbapenem resistance was negligible due to the absence or low level of expression [101]. OXA-23-like enzymes were also encountered in a carbapenem-resistant clinical isolate of the environmental bacterium and opportunistic pathogen *A. baylyi*, where they were associated with a transposon [102], and as well in *A. pittii* [96]. They have been found infrequently integrated into the chromosome of clinical *Proteus mirabilis* (a member of the Family *Enterobacteriaceae*) strains with decreased susceptibility to carbapenems. The genetic environment was identical to the one observed in *A. baumannii*, suggesting the acquisition of the enzyme from a plasmid encoding OXA-23 which was suggested to not be able to replicate in *P. mirabilis* and that was integrated ultimately into the chromosome [52].

*Antibiotic susceptibility and substrate profiles.* In an *E. coli* background OXA-23 confers resistance to penicillins and cephalothin and confers decreased susceptibility to moxalactam and carbapenems. A similar outcome is seen in an *A. baumannii* background, although the MIC values of carbapenems and moxalactam reach levels considered to correspond to clinical resistance [28]. MICs obtained in the presence of inhibitors show that clavulanic acid and tazobactam had some inhibitory activity [52]. It has a low turnover for carbapenems although a very high affinity [28]. The enzyme hydrolyzes effectively the cephalosporin cefotaxime, as well as oxacillin and other penicillins. It shows low turnover of aztreonam and ceftriaxone, and is unable to hydrolyze ceftazidime [13,28,42]. 

*Reported clinical mutants.* Seventeen enzymes share at least 95% sequence identity to OXA-23 (Table 10). OXA-27, a two amino acid derivative, was identified in a clinical *A. baumannii* strain isolated in Singapore in a non-transferrable plasmid. Preliminary kinetic data showed that OXA-27 hydrolyzes penicillins, carbapenems and cephalosporins at a much lower rate. It is sensitive to tazobactam and to a lesser extent clavulanic acid [103]. The chromosome-encoded OXA-102, OXA-103 and OXA-105 derivatives of OXA-23 have six, three and three amino acid substitutions, respectively. They are described as being less susceptible to the carbapenem ertapenem, but not to the carbapenems meropenem or imipenem [101]. OXA-146 differs from OXA-23 by duplication of alanine 220. Unlike OXA-23, OXA-146 effectively hydrolyzes ceftazidime, while keeping a very similar substrate profile for other β-lactams [42].

**Table 10:** OXA-23 like enzymes.

β-Lactamase	Subgroup	First Report Host	Chromosome/Plasmid	GenBank Accession Number	Ref.
OXA-23	OXA-23	*A. baumannii*	Plasmid	AJ132105	[104]
OXA-27	OXA-23	*A. baumannii*	Plasmid	AF201828	[103]
OXA-49	OXA-23	*A. baumannii*	-	AY288523	-
OXA-102	OXA-23	*A. radioresistens*	Chromosome	-	[101]
OXA-103	OXA-23	*A. radioresistens*	Chromosome	-	[101]
OXA-105	OXA-23	*A. radioresistens*	Chromosome	-	[101]
OXA-133	OXA-23	*A. radioresistens*	-	EU571228	[105]
OXA-146	OXA-23	*A. baumannii*	-	FJ194494	-
OXA-165	OXA-23	*A. baumannii*	Plasmid	HM488986	-
OXA-166	OXA-23	*A. baumannii*	Plasmid	HM488987	-
OXA-168	OXA-23	*A. baumannii*	Plasmid	HM488989	-
OXA-167	OXA-23	*A. baumannii*	Plasmid	HM488988	-
OXA-169	OXA-23	*A. baumannii*	Plasmid	HM488990	-
OXA-170	OXA-23	*A. baumannii*	Plasmid	HM488991	-
OXA-171	OXA-23	*A. baumannii*	Plasmid	HM488992	-
OXA-225	OXA-23	*A. baumannii*	-	JN638887	-
OXA-239	OXA-23	*A. baumannii*	-	JQ837239	-

*Location and genetic environment.* OXA-23-like enzymes genes are usually associated with plasmids, although they are also found on the chromosome [52,98,99,106]. The most common genetic environment is the presence of IS*Aba1* elements. The IS*Aba1* insertion sequence is predominantly found in *A. baumannii* and is associated with increased antibiotic resistance as a result of an increased copy number of the enzyme. IS*Aba1* has an important role in the genetic plasticity of *A. baumannii* due to its characteristics as an important mobile element [107,108]. In Tn2006 the OXA-23 gene is bracketed by two identical copies of IS*Aba1* in opposite orientations, while in Tn2008 the downstream IS*Aba1* copy is missing [98,109]. More recently, Tn2009 was described in China: the OXA-23 gene is bracketed by two copies of IS*Aba1*, but they both transcribe in the same direction [110]. Another insertion sequence found associated with the OXA-23 encoding gene is IS*Aba4*, located upstream of the gene, in a transposon denominated Tn2007. The gene is bracketed by IS*Aba4* and a truncated ATPase [109,111]. The hybrid promoter IS*Aba10*-IS*Aba1* is characterized by the insertion of IS*Aba10* into the IS*Aba1* that precedes the OXA-23 gene, with an ATPase located downstream of the gene. Preliminary data suggest that this insertion sequence may confer additional promoter sequences, thereby increasing the expression level of OXA-23 [112].

*Structure determination.* The structure for OXA-23 was determined at several different pH values, and in complex with meropenem. This latter structure is especially interesting as a result of the structural insights it provided with respect to its carbapenemase activity [113]. The structure of an OXA-23 mutant, OXA-146, has also been recently described [42].

### 7.5. OXA-24/40

OXA-24 (OXA-40) was initially identified in the chromosome of a carbapenem-resistant *A. baumannii* clinical strain isolated in 1997 in Spain. Later, the reported sequence for the OXA-24 was revised and it turned out to be identical to the one of OXA-40 [114]. This enzyme is frequently described as “OXA-24/40”.

*Distribution and hosts.* OXA-24/40-like enzymes have worldwide distribution including Europe [115,116], North America [117,115], Asia [16,116,118] and South America [119,120]. Although this enzyme family is predominantly reported in *A. baumannii* strains, OXA-24/40-like enzymes were described in *Acinetobacter haemolyticus* [120], *Acinetobacter pittii* [96], *Acinetobacter baylyi* [121] and *P. aeruginosa* [122].

*Antibiotic susceptibility and substrate profiles.* OXA-24/40 confers resistance to all penicillins and decreases susceptibility to carbapenems when cloned into an *E. coli* background. The MICs for cephalosporins do not change significantly. A similar pattern was seen with OXA-24/40 expressed in *A. baumannii*, but the MICs of the carbapenem antibiotics reached levels that would be considered of resistance [28,114]. The MIC values are not significantly changed by the presence of clavulanic acid, tazobactam and sulbactam [114]. Kinetics performed in the presence of a source of carbon dioxide show a high turnover of oxacillin. The turnover for carbapenems is low, but the affinity is in the nanomolar range, which may explain why the enzyme is able to confer a degree of protection against carbapenems [28]. Kinetics preformed in the absence of a source of carbon dioxide show some activity against penicillins and some cephalosporins, but not against aztreonam, cefepime or cefotaxime. The IC_50_ values for clavulanic acid, tazobactam and sulbactam are 300, 180 and 190 μM, respectively. The enzyme was poorly inhibited by NaCl (IC_50_ of 3 M) [41].

*Reported clinical mutants.* Thirteen OXA-24/40-like enzymes can be sub-divided into four subgroups, each having at least 95% amino acid identity (Table 11.).

OXA-25 (a two amino acid derivative of OXA-24/40) and OXA-26 (a one amino acid derivative of OXA-24/40) were first identified in European strains of *A. baumannii*. Preliminary kinetics demonstrated that they had activity towards penicillins and carbapenems, but not toward cephalosporins [103]. OXA-72, a Gly224Asp mutant of OXA-24/40, was first reported in Thailand (GenBank: AY739646.1) but is now spread worldwide [122,123,124]. OXA-72 confers slightly decreased MIC values in the presence of carbapenems, but keeps the same susceptibility to aztreonam and cefepime as OXA-24/40 [125]. OXA-160 is a Pro227Ser mutant of OXA-24/40 that was first described in the USA. Clones expressing this enzyme are more susceptible to carbapenems than those expressing OXA-24/40, but keep similar levels of susceptibility to aztreonam and cefepime [125].

OXA-143 is a plasmid-borne, 31-amino acid mutant of OXA-24/40. It forms the second subgroup of the OXA-24/40 group. OXA-143 was first identified in 2004 in a Brazilian carbapenem-resistant clinical strain of *A. baumannii*. When cloned into *E. coli* and *A. baumannii* backgrounds, this enzyme conferred decreased susceptibility to penicillins and carbapenems but not to cephalosporins. It hydrolyzes penicillins and carbapenems, although these data may underestimate its substrate profile as they were obtained without a carbon dioxide source. OXA-143 is inhibited by NaCl (IC_50_ of 25 mM). This enzyme was not associated with integrons or insertion sequences. Analysis of the genetic environment suggested that it was acquired through homologous recombination. The current geographic location of this enzyme is essentially limited to Brazil [126]. OXA-231 is a single amino acid derivative of OXA-143 that was recently isolated in Brazil. No data regarding the contribution of the enzyme to β-lactam susceptibility are available [127].

OXA-253 consists of a third subgroup which differs from OXA-143 in 17 amino acids. It has a nearly identical antibiotic susceptibility profile to OXA-143, although it seems to be slightly more active against carbapenem antibiotics [128].

**Table 11:** OXA-24/40 like enzymes.

β-Lactamase	Subgroup	First Report Host	Chromosome/Plasmid	GenBank Accession Number	Ref.
OXA-24/40	OXA-24/40	*A. baumannii*	Chromosome	AF509241	[41]
OXA-25	OXA-24/40	*A. baumannii*	Chromosome	AF201826	[103]
OXA-26	OXA-24/40	*A. baumannii*	Chromosome	AF201827	[103]
OXA-72	OXA-24/40	*A. baumannii*	-	EF534256	[129]
OXA-139	OXA-24/40	*A. baumannii*	-	AM991978	-
OXA-143	OXA-143	*A. baumannii*	Plasmid	GQ861437	[126]
OXA-160	OXA-24/40	*A. baumannii*	-	GU199038	[125]
OXA-182	OXA-182	*A. baumannii*	Plasmid	HM640278	[97]
OXA-207	OXA-24/40	*A. pittii*	-	JQ838185	-
OXA-231	OXA-143	*A. baumannii*	Plasmid	JQ326200	[127]
OXA-253	OXA-253	*A. baumannii*	-	KC479324	-
OXA-255	OXA-253	*A. pittii*	-	KC479325	[96]

*Location and genetic environment.* The genes encoding OXA24/40-like enzymes are found both on the chromosome [114] and on plasmids [120,122] and do not appear associated with insertion sequences or integrons. There is a suggestion that they may be acquired through homologous recombination since they are bracketed by two copies of the same replicase gene [126,127].

*Structure determination.* The structures of the OXA-24/40 apo-enzyme and of two deacylation deficient mutants (in complex with doripenem and oxacillin, respectively) have been determined [54,130,131].

### 7.6. OXA-48

OXA-48 was identified in 2001 in a Turkish strain of multi-drug resistant *Klebsiella pneumoniae*. It was located in a transferrable plasmid and was associated with IS*1999* [43]. OXA-48-like enzymes are now the dominant CHDLs in the *Enterobacteriaceae*. They have not yet been found in *Acinetobacter* or *Pseudomonas*.

*Distribution and hosts.* The OXA-48 group so far has been found exclusively within the *Enterobacteriaceae* (*Citrobacter* and *E. coli*, among others) [132]. The progenitor seems to have derived from a waterborne *Shewanella* species (itself an opportunistic pathogen) in which chromosome-encoded OXA-48-like enzymes are described [133,134]. There are several distinct OXA-48-like genes in the *Enterobacteriaceae* and in diverse *Shewanella* species that are spread worldwide [49,132,135,136,137].

*Antibiotic susceptibility and substrate profiles.* OXA-48 confers resistance to penicillins (with the exception of piperacillin) and decreases the susceptibility to carbapenems, but fails to change the MICs of expanded-spectrum cephalosporins when expressed in *E. coli*. A similar substrate profile is observed in *A. baumannii*, where the enzyme also increases the MIC values of piperacillin and carbapenems to levels of clinical resistance [28]. MIC values obtained in the presence of tazobactam and clavulanic acid did not change [43]. Kinetics obtained in the presence of sodium bicarbonate as a CO_2_ source showed a good turnover for oxacillin and imipenem, but low turnover for other carbapenems. However, the affinity for carbapenems was in the nanomolar range [27]. In the absence of a source of CO_2_ OXA-48 hydrolyzes penicillins and cephalosporins, but not aztreonam [43,138]. The IC_50 _values for clavulanic acid, tazobactam and sulbactam were 16, 1.7 and 50 μM, respectively. OXA-48 is inhibited by NaCl (IC_50_ of 7 mM) [43].

*Reported clinical mutants.* Twelve enzymes could be subdivided into two subgroups (OXA-48 and OXA-54), each with at least 95% sequence identity (Table 12).

**Table 12:** OXA-48 like enzymes.

β-Lactamase	Subgroup	First Report Host	Chromosome/Plasmid	GenBank Accession Number	Ref.
OXA-48	OXA-48	*K. pneumoniae*	Plasmid	AY236073	[43]
OXA-54	OXA-54	*S. oneidensis*	Chromosome	AY500137	[133]
OXA-162	OXA-48	*E. coli*	Plasmid	HM015773	[139]
OXA-163	OXA-48	*E. cloacae*	Plasmid	HQ700343	[140]
OXA-181	OXA-48	*K. pneumoniae*	Plasmid	JN205800	[141]
OXA-199	OXA-48	*S. xiamenensis*	Chromosome	JN704570	[142]
OXA-204	OXA-48	*K. pneumoniae*	Plasmid	JQ809466	[143]
OXA-232	OXA-48	*E. coli*	Plasmid	JX423831	[144]
OXA-244	OXA-48	*K. pneumoniae*	Plasmid	JX438000	[145]
OXA-245	OXA-48	*K. pneumoniae*	Plasmid	JX438001	[145]
OXA-247	OXA-48	*K. pneumoniae*	Plasmid	JX893517	[141]
OXA-370	OXA-48	*Enterobacter sp.*	-	KF900153	-

OXA-162, a Thr213Ala mutant of OXA-48 found in Turkey, confers resistance to penicillins, including piperacillin, while decreasing the susceptibility to cefepime, ceftazidime and carbapenems. Its kinetic properties are very similar to OXA-48 [146]. OXA-163, a one amino acid mutant and four amino acids deletion derivative of OXA-48, is more active against penicillins, expanded-spectrum cephalosporins and aztreonam than OXA-48. It is less active against carbapenems and essentially with no increase in MIC values were observed when the enzyme was expressed in *E. coli*. OXA-163 was more resistant to inhibition by NaCl. The susceptibility to clavulanate and tazobactam remained similar when compared to OXA-48. The turnover and relative affinity values against most penicillins and carbapenems experienced significant decreases, while the opposite was seen for piperacillin and expanded-spectrum cephalosporins [140]. OXA-247, a two amino acid derivative of OXA-163 isolated from the same patient after antibiotic therapy, is more susceptible to expanded-spectrum cephalosporins and aztreonam [147]. OXA-181, identified in *Shewanella siamenensis* in India, and OXA-204 in *K. pneumoniae* in Tunisia, have four and two amino acid substitutions respectively compared to OXA-48. Both have an almost identical susceptibility and kinetic profiles to those of OXA-48. Kinetically, they behave very similarly to OXA-48 [141,143,148]. OXA-232, an Arg214Ser mutant derivative OXA-181, had a similar susceptibility profile to the one of OXA-181, although the MIC values of piperacillin and cephalothin were higher. In the absence of bicarbonate the turnover of ticarcillin, cephalothin and cefepime were higher than OXA-181 [144].

OXA-54, identified in *Shewanella oneidensis*, differs from OXA-48 by 20 amino acids and constitutes a subgroup of the OXA-48-like enzymes. The susceptibility profile conferred by this enzyme is very similar to that of OXA-48, with the exception of piperacillin and cephalothin, for which the MICs are lower. Kinetics obtained in the absence of CO_2_ show comparable activity towards penicillins, carbapenems and cephalosporins but no activity against ceftazidime [133].

*Location and genetic environment.* While the genes encoding OXA-48 enzymes are mostly associated with plasmids, they have been found in the chromosome of diverse species [49,148]. Several genetic environments have been described for these genes. In the first report of OXA-48, the insertion sequence IS*1999* (in Tn*1999*, Tn*1999.2*, Tn*1999.3* and Tn*1999.4*) was found before the gene (under different arrangements) and was suggested to provide promoter sequences and to contribute to the mobilization of the gene. The initial transposon was made of two copies of IS*1999* bracketing the OXA-48 gene and *lysR*. The arrangement of Tn*1999.2* is very similar, but the first IS*1999* is interrupted by IS*1R*. On a third arrangement, Tn*1999.3*, a variant of Tn*1999.2*, a second IS*1R* is found in between the gene for OXA-48 and *lysR*. More recently, Tn*1999.4* was described as a mosaic transposon that also encodes CTX-M-15 β-lactamase [43,149]. The gene of OXA-163 Tn*1999.4* was associated with the IS*Ecl4* element [142], while the ones encoding OXA-204 and OXA-181 were associated with IS*Ecp1* in Tn*2016* and Tn*2013* [142,143].

*Structure determination.* The structure of the apoenzyme of OXA-48 was determined at basic pH [138].

### 7.7. OXA-58

This enzyme was described in 2005 on a plasmid in a multidrug resistant *A. baumannii* strain, isolated in France in 2003 [42].

*Distribution and hosts.* OXA-58-like genes are now disseminated worldwide [10,15,96,115,137,150,151,152,153]. A collection of *Acinetobacter* spp. isolates collected in several countries shows that OXA-58 like enzymes were present in clinical isolates for at least 10 years before their first description [154]. Although OXA-58-like genes/enzymes are most frequently encountered in *A. baumannii* strains, the genes have also been described in other species, such as *Acinetobacter nosocomialis* in Taiwan and Spain [155,156], *A. pittii* in Scotland, Taiwan, Spain [155,157,158], *Acinetobacter haemolyticus* in China [121], and *Acinetobacter junii* and *A. radioresistens* in India [105].

*Antibiotic susceptibility profile and enzyme kinetics.* When cloned in *E. coli* and *A. baumannii* OXA-58 confers resistance to penicillins and cephalothin, and decreased susceptibility to moxalactam and carbapenems, without affecting the susceptibility to other cephalosporins or monobactams [28]. MIC values determined in the presence of tazobactam did not change, but some inhibitory effect was seen with clavulanic acid [44]. In agreement with observations with other CHDLs, this enzyme has a low turnover for carbapenems (especially meropenem, doripenem and ertapenem) but with affinities in the nanomolar range (with the exception of imipenem) [28]. OXA-58 turns over several penicillins and cephalothin fast [28,36]. Inhibition assays performed in the absence of a source of carbon dioxide showed respective IC_50_ values for clavulanic acid, tazobactam and sulbactam of 310, 60 and 2.5 μM. OXA-58 is inhibited weakly by NaCl (IC_50_ of 12 mM) [44].

*Reported clinical mutants.* Four OXA-58 like enzymes are currently known and belong to the same subgroup (Table 13.).

**Table 13:** OXA-58 like enzymes.

β-Lactamase	Subgroup	First Report Host	Chromosome/Plasmid	GenBank Accession Number	Ref.
OXA-58	OXA-58	*A. baumannii*	Plasmid	AY665723	[44]
OXA-96	OXA-58	*A. baumannii*	Plasmid	DQ519090	[159]
OXA-97	OXA-58	*A. baumannii*	Plasmid	EF102240	[153]
OXA-164	OXA-58	*A. baumannii*	plasmid	GU831575	[160]

OXA-164, a Phe114Leu mutant of OXA-58, seems to be more sensitive to meropenem, but not imipenem, than OXA-58 [160]. OXA-97, a Ala54Gly mutant of OXA-58 isolated in Tunisia, confers the same antimicrobial susceptibility profile as OXA-58 [153]. 

*Location and genetic environment*. OXA-58-like enzymes encoding genes are usually associated with plasmids in *Acinetobacter* species. The analysis of their genetic environment has shown that they are usually bracketed by two IS*Aba3* insertion sequences, upstream and downstream of the gene [44]. Several arrangements, however, have been described. All have IS*Aba3* downstream of the gene, but upstream there is variability. Combinations of IS*Aba3* with IS*Aba1*, IS*Aba2*, IS*Aba125*, IS*Aba825*, IS*Aba8*, IS*1008* and IS*1006* are described [81,152,156,159,161,162,163]. Poirel *et al*., give a detailed analysis of the genetic structures surrounding OXA-58-like genes [159]. These sequences have promoter sequences, and when two insertion sequences are together they may generate a hybrid promoter which can increase gene expression [162,163,164]. There have also been reports of multiple copies of the OXA-58 gene in the same plasmid, which also contributes to an increased copy number of the enzyme [165].

*Structure determination.* The structure of the OXA-58 apoenzyme recently was described [27].

### 7.8. OXA-134

*Distribution and hosts.* OXA-134 is found in diverse *Acinetobacter* species including *A. baumannii*, *A.* *lwoffii* and *A. schindleri* [96]. Its origin has been linked to *Acinetobacter lwoffii*, a human commensal and occasional pathogen [30]. *A. schindleri* also encodes an OXA-134-like β-lactamase, but it seems to be from a different cluster [166]. The only members of this group that have been found in plasmids belong to the OXA-235 subgroup, and are the focus of our discussion [167].

*Antibiotic susceptibility and substrate profiles.* OXA-235 is the representative of the OXA-235 subgroup and was responsible for a decreased susceptibility to carbapenems when expressed in *A. baumannii* and *A. baylyi*. Kinetically, it showed a fast turnover of oxacillin, but surprisingly the turnover for ampicillin and carbapenems was very low. While a slow turnover for carbapenems is not unexpected, the reduced value for ampicillin turnover may be due to the lack of a source of carbon dioxide in the assay [167].

*Reported clinical mutants.* There are several clinical mutants comprising three distinct subgroups (Table 14.), but only the only plasmid encoded protein that has been studied is OXA-235.

**Table 14:** OXA-134 like enzymes.

β-Lactamase	Subgroup	First Report Host	Chromosome/Plasmid	GenBank Accession Number	Ref.
OXA-134	OXA-134	*A. lwoffii*	Chromosome	HQ122933	[31]
OXA-186	OXA-134	*A. lwoffii*	Chromosome	-	[31]
OXA-187	OXA-187	*A. lwoffii*	Chromosome	-	[31]
OXA-188	OXA-187	*A. lwoffii*	Chromosome	-	[31]
OXA-189	OXA-187	*A. lwoffii*	Chromosome	-	[31]
OXA-190	OXA-187	*A. lwoffii*	Chromosome	-	[31]
OXA-191	OXA-187	*A. lwoffii*	Chromosome	-	[158]
OXA-235	OXA-235	*A. baumannii*	Plasmid	JQ820240	[167]
OXA-236	OXA-235	*A. baumannii*	Plasmid	JQ820242	[167]
OXA-237	OXA-235	*A. baumannii*	Plasmid	JQ820241	[167]

*Location and genetic environment*. In *A. baumannii*, the OXA-235 subgroup genes are associated with two copies of IS*Aba1* [167].

*Structure Determination*. No structure of any of members of the OXA-134 group is available.

### 7.9. Other OXA Groups

Several other less important groups have been described (Table 15).

*OXA-9.* This enzyme was initially described associated with a transposon in a *K. pneumoniae* strain [168]. The enzyme has been described worldwide, and is considered a narrow spectrum β-lactamase [10] which is not inhibited by sodium chloride [169].

*OXA-18.* OXA-18, an extended-spectrum Class D β-lactamase, was described in France in 1995 in a *P. aeruginosa* isolate [32]. Later reports demonstrate its spread [170,171]. It increases the MIC values of penicillins, expanded spectrum cephalosporins and aztreonam. It is sensitive to inhibition by clavulanic acid. The kinetic data correlate with the MIC data, and the IC_50_ values for the inhibitors are in the nanomolar range [32]. This enzyme had a chromosomal location, where it was bracketed by two copies of the insertion sequence IS*CR19* [170].

*OXA-20.* OXA-20 is a narrow-spectrum enzyme identified in *P. aeruginosa* in France in 1995 which has disseminated in Europe and is now found in *A. baumannii* [10]. OXA-20 is located in the chromosome in an integron that encodes an aminoglycoside acetyltransferase. When cloned in *E. coli* OXA-20 increased the MIC values for penicillins, cephalothin and imipenem but not those of extend-spectrum cephalosporins. It was inhibited by clavulanic acid. OXA-20 hydrolyzes penicillins, cephalothin and aztreonam [33]. One mutant of this enzyme, OXA-37, is known [172].

*OXA-45.* OXA-45 was initially identified in a multidrug resistant *P. aeruginosa* isolate from Texas. No other OXA enzymes shares more than 80% sequence identity with OXA-45. It has an expanded-spectrum profile: it confers resistance to ceftazidime and ampicillin and increases the MIC values of piperacillin and of expanded-spectrum cephalosporins. The MIC values of carbapenems were not significantly changed. OXA-45 is sensitive to clavulanic acid [173].

*OXA-51.* OXA-51 was described for the first time in 2004, in carbapenem-resistant strains isolated in Argentina between 1993–1994 [174]. This is a predominantly chromosome-encoded CHDL from *Acinetobacter baumannii* that has been recently described as associated with mobile elements in several *Acinetobacter* species [175,176]. Although there are multiple OXA-51 mutants, little is known about their activity. Members of this family behave as poor penicillinases and very weak carbapenemases, without activity against cephalosporins. They are also resistant to inhibition by clavulanic acid and tazobactam [29,173,177].

*OXA-198.* OXA-198 was identified on a Class 1 integron in a *P. aeruginosa* strain resistant to several antimicrobials. It decreased susceptibility to penicillins, but it did not change the MICs of cephalosporins. It was resistant to tazobactam [178].

*LCR-1.* LCR-1 was first described in 1982 in *P. aeruginosa* strain from the United States. It hydrolyzed various penicillins, but neither cefotaxime nor imipenem [179,180]. Another member of this group having a similar kinetic behavior, NPS-1, was also found in *P. aeruginosa* in the United Kingdom. When expressed in *P. aeruginosa* LCR-1 it did not change the susceptibility to cephalosporins, monobactams or carbapenems [181].

**Table 15:** Other OXA groups.

β-Lactamase	Subgroup	Spectrum	First Report Host	Chromosome/Plasmid	GenBank Accession Number	Ref.
OXA-9	-	Narrow	*K. pneumoniae*	Plasmid	M55547	[168]
OXA-18	-	Extended	*P. aeruginosa*	Chromosome	U85514	[32]
OXA-20	-	Narrow	*P. aeruginosa*	Chromosome	AF024602	[33]
OXA-45	-	Expanded	*P. aeruginosa*	Plasmid	AJ519683	[173]
OXA-51	-	Carbapenemase	*A. baumannii*	Chromosome	AJ309734	[174]
OXA-198	-	Narrow	*P. aeruginosa*	Plasmid	HQ634775	[178]
LCR-1	-	Narrow	*P. aeruginosa*	Plasmid	Q00983	[89]

## 8. Future Prospects/Conclusions

It is difficult to determine just how widespread the problematic acquired Class D β-lactamases have become. Although the epidemiologic studies regarding the most characterized enzymes are very comprehensive, little is known about the less important enzymes. Traditionally, standard sets of oligonucleotides are used to identify enzymes in the clinical environment. These sets are chosen so as to identify the most common enzymes. However, this methodology fails to identify those enzymes presumed (possibly incorrectly) to be less important, as well as new enzyme variants. Since the existence of several resistance determinants in the same bacterium is common, a positive identification does not mean that the enzyme responsible for a phenotype of resistance has been identified.

A major concern regarding the study of Class D β-lactamases is the methodology employed for the *in vitro* study of their kinetics. The importance of including a source of carbon dioxide in the reaction buffer when performing kinetic studies has been well established during the years. However, studies continue to report kinetics performed under non-appropriate conditions. The entry of such data into the literature poses a major problem, as a critical comparison between the results obtained by different groups is made difficult, if not impossible. The use of standardized methods is a critical determinant of the value of the research.

Another concern is the nomenclature for the Class D β-lactamases. While the current numerical nomenclature communicates the enzyme being described, the numerical nomenclature fails to communicate the character of the enzyme. With more than 400 OXA enzymes described at this moment, a numerical nomenclature that clearly identifies interrelationships between enzymes, as has developed within the other β-lactamase classes (such as the TEM-prefix for Class A, the VIM-prefix for Class B, and the ADC-prefix for Class C) would have value. While renaming all the Class D is not possible (and would itself be a source of problems), a simple and understandable numbering scheme is possible. We suggest a modified numbering scheme that introduces between the OXA name and the current number the group number. For example, OXA-51 would become OXA_51_-51 and OXA-69 would become OXA_51_-69, identifying these two enzymes as members of the same group. While in the beginning there would be inevitably gaps, in time these gaps would fill. The cutoff for the determination of the enzymes groups could be redefined from the 80% value we use on this review.

Class D enzymes have made their way into clinical practice and we will have to account for them for the near future. The prolific studies of recent years have widened in what we know about these enzymes. However, there is the critical need for further studies on how these enzymes develop resistance to clinically important β-lactams. The new inhibitors and new β-lactams that are being developed have promise. The continuing investment into resources to understand and to combat these enzymes is critical, lest we fall even further behind in this microscopic challenge.
